# Optimising Camera–ChArUco Geometry for Motion Compensation in Standing Equine CT: A CT-Motivated Benchtop Study

**DOI:** 10.3390/s26041310

**Published:** 2026-02-18

**Authors:** Cosimo Aliani, Cosimo Lorenzetto Bologna, Piergiorgio Francia, Leonardo Bocchi

**Affiliations:** 1 Department of Information Engineering, University of Florence, Via di Santa Marta 3, 50139 Florence, Italy; cosimo.aliani@unifi.it (C.A.); leonardo.bocchi@unifi.it (L.B.); 2 Epica Imaginalis, Via Rodolfo Morandi 13/15, 50019 Florence, Italy; c.lorenzetto@imaginalis.it

**Keywords:** computed tomography, equine, ChArUco, camera–marker geometry, motion compensation

## Abstract

Standing equine computed tomography (CT) acquisitions are susceptible to residual postural sway, which can introduce view-inconsistent motion and degrade image quality. External optical tracking based on ChArUco fiducials is a promising, low-cost strategy to enable projection-wise motion compensation, yet quantitative guidance on how camera–marker geometry affects pose-estimation performance remains limited. This CT-motivated benchtop study characterizes how the relative camera–ChArUco configuration influences both the accuracy (bias with respect to ground truth) and the precision (repeatability) of pose estimates obtained from RGB images using OpenCV ChArUco detection and reprojection-error minimization to estimate the rigid camera-to-board transformation. Controlled experiments systematically varied acquisition protocol (continuous repeated estimates at fixed pose versus cyclic repositioning), viewing angle over a wide angular range at two working distances, and camera-to-board distance over multiple depth settings. Ground truth for angular configurations was defined by a stepper-motor rotation stage, while distance ground truth was obtained by ruler measurements. Performance was summarized via mean absolute error and standard deviation across repeated measurements, complemented by variance-based statistical testing with multiple-comparison correction. Cyclic repositioning did not yield evidence of increased variability relative to continuous acquisitions, supporting view-by-view sampling. Viewing angle induced a consistent accuracy–precision trade-off for rotations: frontal views minimized mean error but exhibited higher variability, whereas oblique views reduced jitter at the expense of increased bias. Increasing working distance reduced repeatability, most prominently for depth-related components. Overall, these findings provide pre-clinical guidance for selecting camera/marker placement (moderately oblique viewpoints, limited working distance, sufficient image footprint) before in-scanner and in-vivo validation for standing equine CT motion compensation.

## 1. Introduction

Computer Vision (CV) focuses on the automatic extraction, analysis, and interpretation of meaningful information from single images or sequences of images, and relies on theoretical frameworks and algorithms for autonomous visual understanding [1]. A long-standing challenge concerns three-dimensional (3D) reconstruction from two-dimensional (2D) images, which arises in multiple domains—robotics [2,3], medicine [4,5], and virtual reality [6,7]—and stems from the loss of depth information when projecting a 3D scene onto the image plane ($f\;:R^{3}\;\to \;R^{2}$). Approaches such as photogrammetry, stereo vision (SV), and structure-from-motion (SfM) address this task by detecting distinctive features (keypoints), establishing correspondences across views, and combining image evidence with camera parameters to infer 3D coordinates for scene points.

In many of these pipelines, accurate and stable camera pose estimation is a key determinant of reconstruction quality. Physical fiducials with known geometry are widely used to stabilize pose: ArUco markers [8,9] offer robust, uniquely identifiable square patterns but limited corner refinement; chessboards enable subpixel-accurate corner localization yet require full visibility and are less tolerant to occlusions [10,11]. ChArUco boards combine these strengths by embedding ArUco markers within a chessboard grid, achieving reliable detection under partial occlusion together with high-precision corner refinement via interpolated chessboard corners [11]. Thanks to the known layout, a single image suffices to estimate the rigid transformation between camera and board, typically decomposed into rotation (yaw, pitch, roll) and position components ($T_{x}$, $T_{y}$, $T_{z}$).

A setting where precise, projection-wise pose tracking is particularly valuable is computed tomography (CT) of standing horses. Dedicated systems for standing acquisitions of the head and distal limbs allow imaging of sedated equine patients in a weight-bearing position, avoiding the risks of general anaesthesia while preserving diagnostic performance [12,13,14,15,16]. However, residual postural sway during gantry rotation induces view-inconsistent motion that manifests as blur and streak artifacts and may reduce confidence in the assessment of fine osseous detail [14,15]. To mitigate such motion, previous work has mainly relied on mechanical restraints and acquisition protocols, while software-based motion compensation is still uncommon in routine equine imaging and lacks quantitative design guidelines.

**Figure 1:**
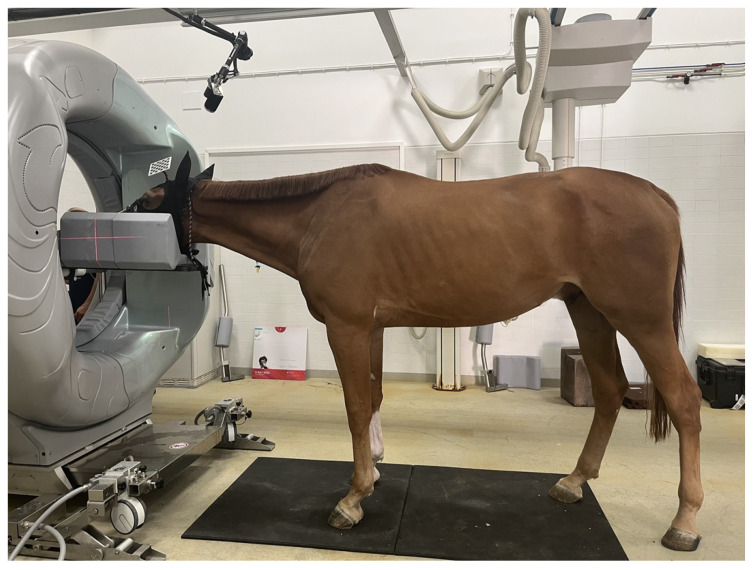
Representative photograph illustrating the intended standing equine cone beam computed tomography (CBCT) head-imaging workflow. A ChArUco board is rigidly attached to the horse’s head and observed by an RGB camera for external pose tracking in the target clinical context. This image is provided for illustrative purposes only; no data from the depicted animal/procedure were acquired or used in the present study.

As shown in Figure 1, a practical strategy is to rigidly mount a lightweight ChArUco board near the region of interest and observe it with an auxiliary RGB camera during acquisition. Each frame provides a board pose that, via a fixed extrinsic calibration to the scanner, can be mapped to object motion for projection-wise motion compensation. In this context, the camera–marker geometry—specifically, viewing angle and working distance—directly affects both pose accuracy (bias with respect to ground truth) and pose precision (variance/noise), and thus the effectiveness of motion correction.

By contrast, in human CT, several prototype systems already exploit external motion sensing for projection-wise motion correction. Optical trackers, inertial sensors, and RGB-D cameras have been used to monitor patient motion during helical or cone-beam scans and to warp the projections accordingly, recovering image quality that approaches that of motion-free acquisitions [17,18,19]. Low-cost Intel RealSense devices in particular have been characterized for clinical motion tracking and CT perfusion motion correction, showing millimetric pose accuracy in realistic scanner environments [20,21,22]. These developments motivate the use of compact RGB or RGB-D modules as external motion sensors in the standing equine CT workflow.

Recent work also highlights complementary directions to handle motion and tracking uncertainty. On the motion-compensation side, projection-driven and learning-based strategies have been proposed in CT to estimate and compensate motion directly from image/projection data, reducing reliance on external sensors in some settings [23,24,25]. On the tracking side, the fiducial-marker literature has progressed beyond standard OpenCV pipelines: deep-learning-based detection has been introduced to improve robustness under adverse illumination and imaging conditions [26], multi-marker fusion with spatial/temporal outlier rejection has been shown to improve both accuracy and precision especially at large tilt angles [27], and recent benchmarking studies report that camera type and imaging geometry can materially affect fiducial-based pose stability [28]. These advances reinforce that both the tracking algorithm and the camera–marker geometry contribute to performance.

Although ChArUco boards are already adopted across diverse applications (e.g., RGB-D fusion [29], omnidirectional camera calibration [30], and robust detection in challenging conditions [31]), the quantitative relationship between pose-estimation accuracy/precision and the relative camera–board configuration remains underexplored. In particular, there is limited evidence on how viewing angle and camera-to-plane distance shape the error with respect to a known ground truth and the noise of estimated rotations and positions, despite the practical impact on reconstruction quality in motion-aware workflows and on the design of standing equine CT setups.

The present study addresses this gap through a controlled experimental analysis that systematically varies camera-to-plane angles and camera-to-plane positions, acquiring repeated measurements to characterize how pose accuracy and precision change with viewpoint. The resulting trends are interpreted as CT-motivated, pre-clinical guidance to inform camera/marker placement prior to future in-scanner and in-vivo validation, thereby limiting iterative optimization on live animals.

## 2. Materials and Methods

**Figure 2:**
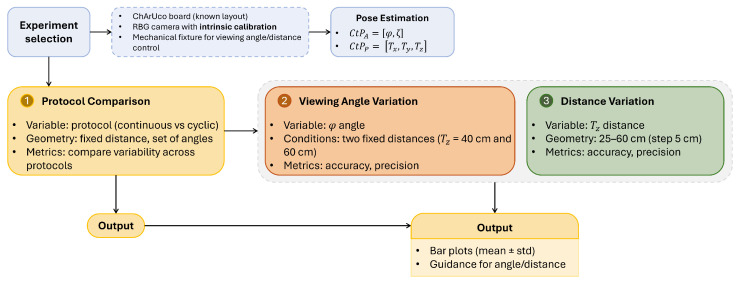
Flowchart of the experimental workflow and study design. A common pose-estimation pipeline (setup, acquisition, and ChArUco-based pose estimation) is applied across three experiments: protocol comparison (continuous vs. cyclic), viewing-angle variation at fixed working distances, and working-distance variation. Outputs include accuracy/precision metrics (mean ± standard deviation) used to derive practical guidance on camera–board geometry.

This work adopts a controlled benchtop experimental design to quantify how camera–ChArUco geometry affects pose-estimation accuracy and precision. Repeated pose estimates were collected under identical conditions while systematically varying, separately, the camera-to-plane viewing angle and the camera-to-board working distance (and associated translations). Ground truth for angular configurations was defined by the commanded stepper-motor rotation stage (1° increments), whereas ground truth for distance changes was obtained via ruler measurements. For each geometric condition, pose was estimated from RGB images using OpenCV ChArUco detection and perspective-*n*-point (PnP), and performance was summarized using mean absolute error with respect to ground truth (accuracy) and the standard deviation across repeated measurements (precision). An overview of the full implementation workflow and the three experimental branches is provided in Figure 2.

### 2.1. ChArUco Board

**Figure 3:**
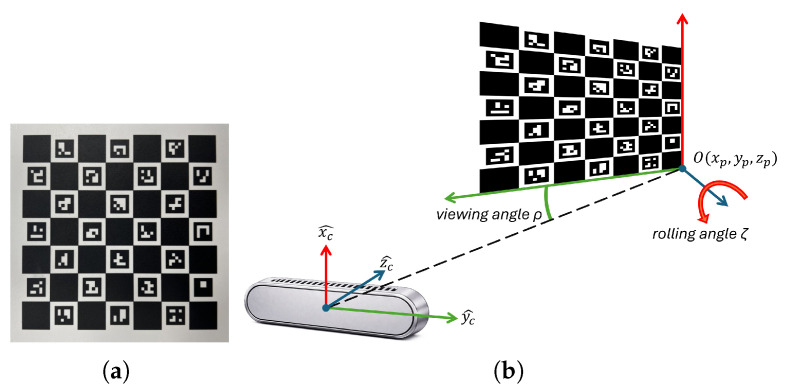
(**a**) ChArUco board used during the experiments. (**b**) Camera and ChArUco reference systems and definition of $CtP_{A}$ and $CtP_{P}$. ChArUco’s origin $O(x_{p},y_{p},z_{p})$ is expressed in the camera reference system.

The ChArUco board used in this study, shown in Figure 3a, consists of a 7 × 7 chessboard grid with 24 unique ArUco markers placed inside the white squares. Each chessboard square measures 15 mm per side, while each ArUco marker measures 10 mm per side. The relative pose between the camera and the board was parameterized by five scalar quantities:

- **Camera-to-plane angles (**${\mathrm{CtP}}_{A}$**):** $CtP_{A}=\left[\varphi ,\zeta \right]$, where φ is the angle between the camera optical axis (${\hat{z}}_{c}$) and the board normal vector $\hat{n}$, and ζ is the in-plane rotation around $\hat{n}$.
- **Camera-to-plane positions (**${\mathrm{CtP}}_{P}$**):** $CtP_{P}=\left[T_{x},T_{y},T_{z}\right]$, describing the position of the board origin expressed in the camera reference system.

To improve clarity, parameters defining both $CtP_{A}$ and $CtP_{P}$ are depicted in Figure 3b.

Both the detection of the ChArUco board and the camera pose estimation were performed using the open-source OpenCV library [32], in conjunction with the Qt Creator development environment (version 5.12.6) and the C++ programming language.

### 2.2. RGB Camera

The RGB camera employed in this study is the RGB module of the Intel RealSense D435 3D camera (Intel Corporation, Santa Clara, CA, USA). Its key specifications are summarized in Table 1.

### 2.3. Mechanical Systems for Camera or ChArUco Handling

Once rigidly attached to the horse, the ChArUco target is expected to undergo predominantly translational excursion $T_{z}$ (towards/away from the observing camera) and changes in viewing angle φ, rather than in-plane rotation ζ about the board normal. This reflects the residual postural sway of the sedated standing subject around its resting stance.

**Figure 4:**
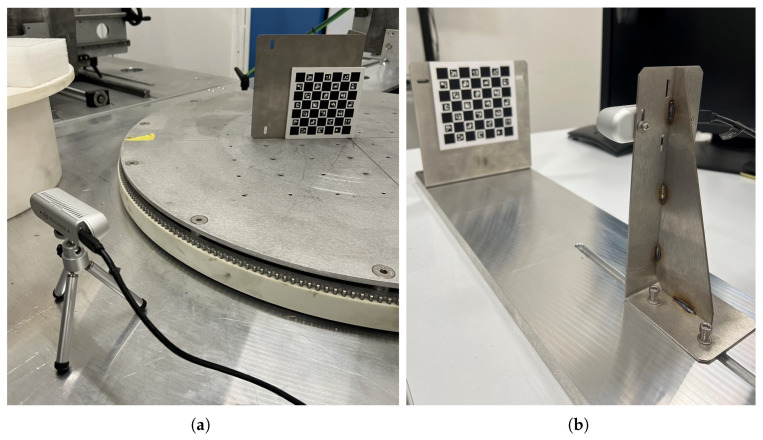
(**a**) Setup used during the test for the evaluation of the viewing angle φ parameter. The camera was placed on a tripod to ensure stability. (**b**) Setup used during the test for the evaluation of the $T_{z}$ parameter.

Thus, the experimental setups were designed to independently vary either the viewing-angle φ or the position along the z-axis $T_{z}$ between the ChArUco board and the camera, while monitoring the remaining pose parameters to verify that off-axis positions and ζ remained negligible:

- **Viewing angle φ setup:** as shown in Figure 4a, this setup consists of a steel mechanical wheel that rotates precisely about its axis in 1° increments and is driven by a stepper motor. Two orthogonal grooves intersecting at the wheel’s centre enable precise placement of the ChArUco board such that its coordinate origin coincides with the wheel’s axis of rotation. This alignment is critical to minimize undesired translational components and rotation about ζ during wheel motion.
- **$T_{z}$ position setup:** as shown in Figure 4b, this setup uses a 1-m-long steel rail with a central horizontal groove. The ChArUco board is fixed to an L-shaped bracket and mounted orthogonally at one end of the base. The camera is attached to a separate L-shaped bracket and positioned to face the ChArUco board. By sliding the bracket along the groove, the distance between the camera and the ChArUco board is adjusted along the *z*-axis while minimizing off-axis motion.

For the viewing-angle experiments, the wheel was controlled via its dedicated software, which allowed setting angular positions in 1° increments with respect to a reference configuration defined as φ=0°. Accordingly, the ground-truth rotation $\varphi_{\mathrm{GT}}$ was taken as the wheel angle set in the control software. To reduce the effect of mechanical backlash, target angles were approached consistently using the same rotation direction.

For the distance experiments, the ground-truth camera-to-plane distance $T_{z,\mathrm{GT}}$ was measured using a ruler. Measurements were taken between the camera reference point and the ChArUco board plane (as defined in Figure 3b) at each tested position, and recorded with the ruler resolution.

Pose accuracy was quantified as the absolute error with respect to ground truth, e.g., $e_{\varphi }=\left|\varphi -\varphi_{\mathrm{GT}}\right|$ and $e_{T_{z}}=\left|T_{z}-T_{z,\mathrm{GT}}\right|$, while precision was quantified as the variability (e.g., standard deviation) across repeated measurements under identical conditions.

### 2.4. Measurements Acquisition Procedure

Multiple tests were conducted to evaluate the accuracy of camera pose estimation relative to the ChArUco marker. In each test, the camera was initially positioned relative to the ChArUco board, and the pose parameters $CtP_{A}=\left[\varphi ,\zeta \right]$ and $CtP_{P}=\left[T_{x},T_{y},T_{z}\right]$ were acquired.

Before each run, an initial reference pose was recorded at the start position: $CtP_{A_{0}}=\left[\varphi_{0},\zeta_{0}\right]$ and $CtP_{T_{0}}=\left[T_{x_{0}},T_{y_{0}},T_{z_{0}}\right]$. These reference values were subtracted from each subsequent measurement to describe rotation/position increments rather than absolute pose. Repeatability and drift were quantified across repeated runs using statistics of these relative pose trajectories. Absolute accuracy was also evaluated as the error with respect to the known ground truth defined for each test.

#### 2.4.1. Continuous and Cyclic Acquisitions

The first experiment aimed to assess the noise introduced by the camera and by the mechanical movement system. For this test, the camera was fixed at a distance of $T_{z}$ = 40 cm from the ChArUco board, and the viewing angle φ was varied using the mechanical wheel. The tested angles were [45°, 25°, 5°, 0°, −5°, −25°, −45°], using two acquisition methods:

- **Continuous acquisitions:** To evaluate noise introduced solely by the camera, each pose was estimated 10 times at each angle before rotating the wheel to the next angle.
- **Cyclic acquisitions:** To assess the influence of the mechanical system, only one pose was estimated at each angle. After reaching −45°, the wheel was rotated back to 45° in a single motion, and the process was repeated 10 times in total.

After each acquisition, the standard deviation of all five pose parameters was computed to compare the consistency of the two acquisition approaches.

This comparison is motivated by projection-wise motion tracking in CT: during an acquisition, the tracker delivers one pose estimate per projection while the gantry (or the object) moves between successive views. “Continuous” acquisitions isolate the intrinsic measurement noise of the camera/estimator at a fixed viewpoint (no repositioning). “Cyclic” acquisitions mimic repeated view-by-view sampling across angles and re-positioning, therefore capturing additional variability introduced by the mechanical motion (e.g., backlash, settling, and repeatability of the rotation wheel), which is analogous to view-to-view perturbations during a CT sweep.

#### 2.4.2. Viewing Angle Variation

The second experiment evaluated how viewing angle estimation accuracy varies with the viewing angle. Angles ranging from 60° to −60° were tested: [60°, 50°, 45°, 40°, 30°, 20°, 10°, 0°, −10°, −20°, −30°, −40°, −45°, −50°, −60°]. The ChArUco board was aligned with the rotation axis of the mechanical wheel, and the camera was placed at fixed distances of $T_{z}$ = 40 cm and $T_{z}$ = 60 cm. At each angle and distance, 20 pose estimations were acquired to ensure statistical robustness.

#### 2.4.3. Z-Axis Position Variation

The third experiment focused on how the position along the z-axis affects the estimation of the $T_{z}$ parameter. Camera poses were estimated at distances ranging from $T_{z}$ = 25 cm to $T_{z}$ = 60 cm, in 5 cm increments ($\Delta T_{z}$ = 5 cm). As in the previous test, each position was measured 20 times to support statistical analysis.

### 2.5. Pose Estimation Model and Performance Metrics

To provide theoretical support for the implementation, the pose-estimation model and the adopted performance metrics are formalized below.

Let $x_{i}={\left[X_{i},Y_{i},Z_{i}\right]}^{⊤}$ be a 3D point on the ChArUco board expressed in the board frame. Under a pinhole camera model, its image projection $u_{i}={\left[u_{i},v_{i},1\right]}^{⊤}$ satisfies

(1) $$s\;u_{i}=K\;\left(Rx_{i}+t\right),$$

where *s* is a projective scale factor (proportional to the point depth in the camera frame), K is the intrinsic calibration matrix, and (R,t) is the rigid transform from board to camera coordinates, with R mapping vectors from the board frame to the camera frame.

Given detected 2D corners ${\tilde{u}}_{i}$, the pose is obtained by solving a perspective-*n*-point (PnP) problem, i.e.,

(2) $$\left(\hat{R},\hat{t}\right)=\arg\min_{R,t}\sum_{i=1}^{N}{∥{\tilde{u}}_{i}-\pi \left(K(Rx_{i}+t)\right)∥}^{2},$$

where π(·) denotes perspective division, and $x_{i}$ denotes the corresponding 3D points in the board frame.

The estimated translation is directly given by $\hat{t}={\left[{\hat{T}}_{x},{\hat{T}}_{y},{\hat{T}}_{z}\right]}^{⊤}$ (board origin expressed in the camera frame). Let ${\hat{n}}_{c}=\hat{R}\;n_{p}$ be the board normal expressed in the camera frame, with $n_{p}={\left[0,0,1\right]}^{⊤}$. The viewing angle is then

(3) $$\hat{\varphi }=\arccos\left(\frac{{\hat{n}}_{c}^{⊤}{\hat{z}}_{c}}{∥{\hat{n}}_{c}∥\;∥{\hat{z}}_{c}∥}\right),$$

where ${\hat{z}}_{c}={\left[0,0,1\right]}^{⊤}$ is the camera optical axis. The in-plane rotation $\hat{\zeta }$ is obtained from the estimated rotation matrix $\hat{R}$ as the rotation about the board normal ${\hat{n}}_{c}$, i.e., the in-plane (roll) component of the board orientation.

## 3. Results

In the following subsections, the results for each of the three conducted tests will be presented.

In all bar plots, bar height represents the mean absolute error with respect to ground truth, while error bars indicate the standard deviation across repeated measurements under the same condition.

### 3.1. Continuous vs. Cyclic Acquisition

**Figure 5:**
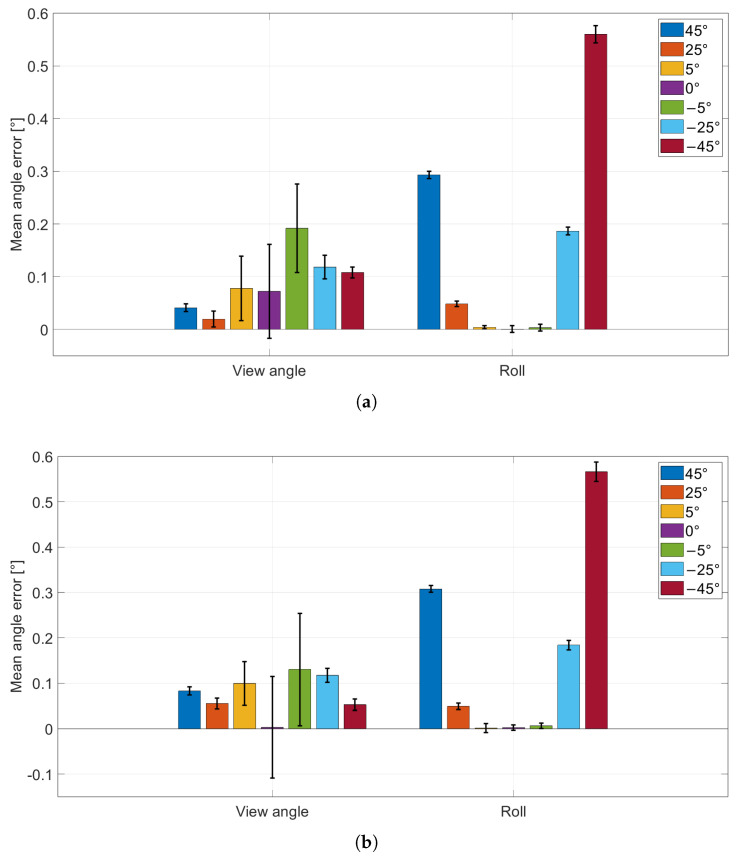
$CtP_{A}$ parameters absolute error (bar height; viewing angle φ and roll ζ) with standard deviation (error bars) for each wheel position. (**a**) Continuous acquisition; (**b**) cyclic acquisition.

**Figure 6:**
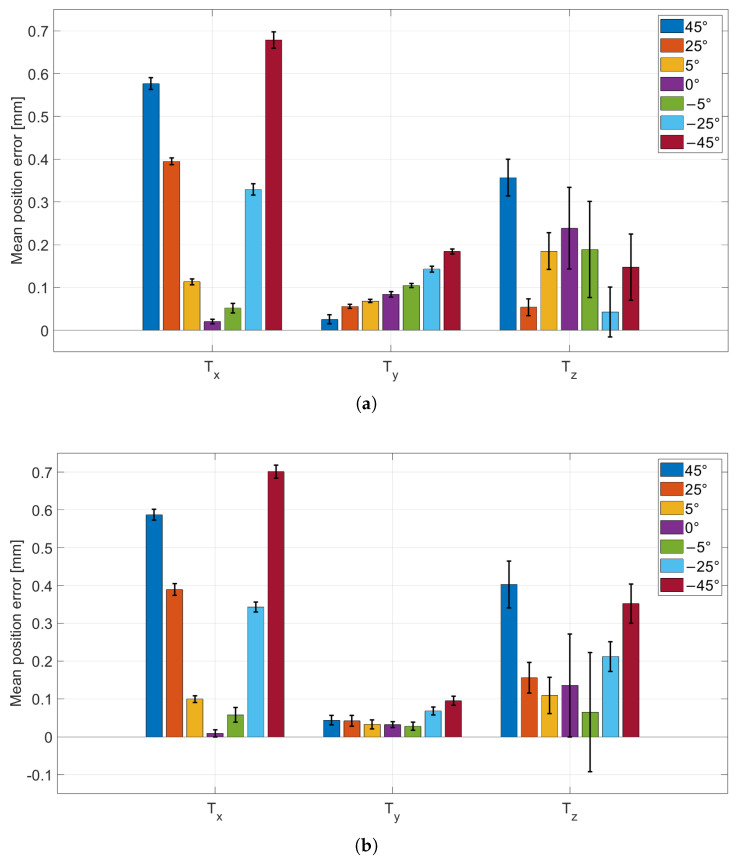
$CtP_{P}$ parameters absolute error (bar height; $T_{x}$, $T_{y}$, $T_{z}$) with standard deviation (error bars) for each wheel position. (**a**) Continuous acquisition; (**b**) cyclic acquisition.

The results of this test are reported in Figure 5 and Figure 6. Figure 5a,b show the mean absolute errors of the $CtP_{A}$ parameters for the continuous and cyclic protocols, respectively. Figure 6a,b show the corresponding mean absolute errors for the $CtP_{P}$ parameters.

The mechanical wheel provided controlled viewpoint changes in 1° increments through stepper-motor actuation. The comparison between continuous and cyclic acquisitions was used to verify that repeated repositioning of the wheel does not introduce additional variability beyond the intrinsic pose-estimation noise.

To assess whether the two acquisition protocols differ in a statistically meaningful way, Levene’s test for equality of variances was applied to the error distributions obtained with the continuous and cyclic protocols. For the formal statistical comparison, three representative wheel positions were selected (−45°, 0°, +45°), corresponding to the two most oblique views and the frontal configuration. This choice captures the extremes of the investigated angular range (i.e., a worst-case condition for perspective effects) as well as the frontal case, while limiting the number of multiple comparisons. Intermediate angles were analyzed descriptively in the plots and are expected to lie within the bounds defined by these representative configurations. For each selected wheel position, a one-to-one comparison was performed for each parameter (i.e., φ, ζ, $T_{x}$, $T_{y}$, $T_{z}$), resulting in 15 comparisons in total. Levene’s test was selected because the mean absolute error can be influenced by systematic effects (e.g., small setup misalignments or mechanical offsets), whereas the variance (and thus the standard deviation) more directly reflects repeatability and intrinsic measurement noise, which is the key aspect when evaluating whether cyclic repositioning introduces additional variability. Across the 15 comparisons, only $T_{y}$ at −45° showed a nominally significant result (p=0.03783). A Bonferroni correction for multiple comparisons was applied, setting the corrected significance level to $\alpha_{B}=0.05/15\approx 0.0033$; under this threshold, no comparison was significant. Therefore, no evidence was found that cyclic repositioning systematically changes measurement variability compared to the continuous protocol, and subsequent experiments are reported using the continuous acquisition method only.

### 3.2. Influence of Viewing Angle on Pose Estimation

**Figure 7:**
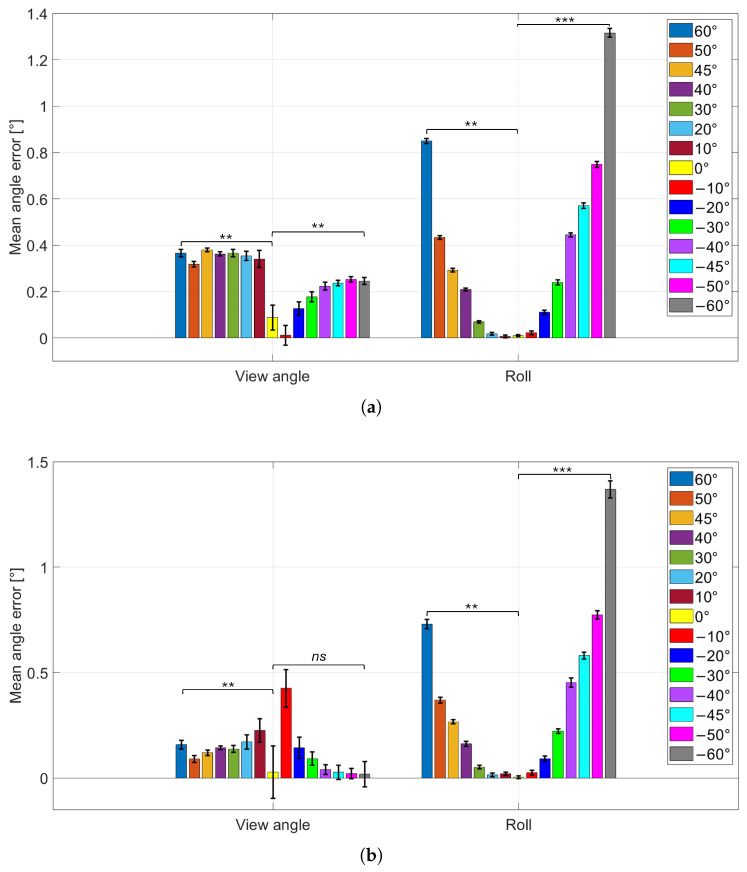
$CtP_{A}$ parameters absolute error (viewing angle φ and roll ζ) for each wheel position. (**a**): $T_{z}$ parameter fixed at 40 cm; (**b**): $T_{z}$ parameter fixed at 60 cm. Statistical significance is indicated using symbols: *ns* for not statistically significant, ** for p<0.001, and *** for p<0.0001.

**Figure 8:**
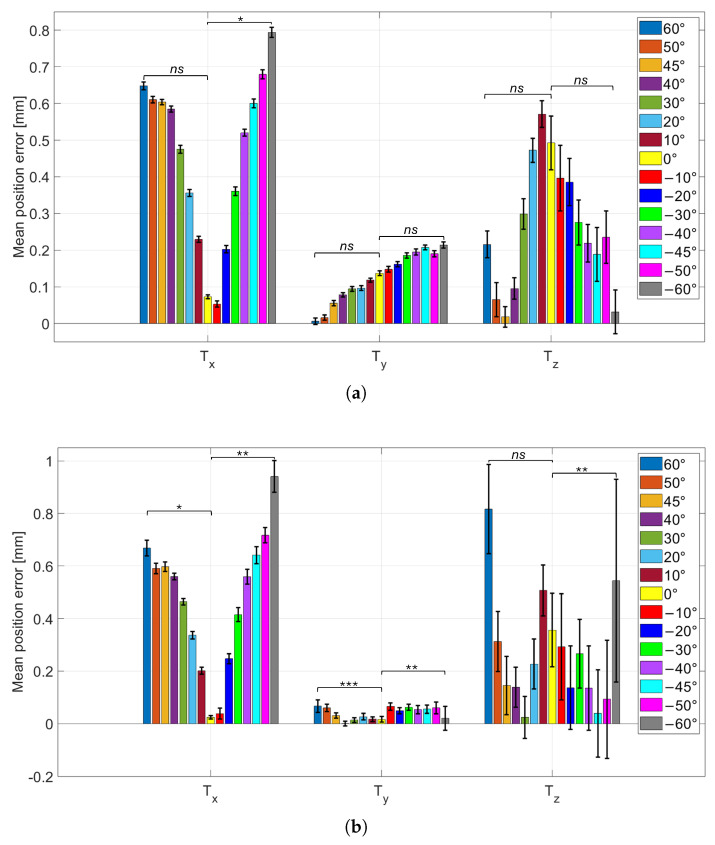
$CtP_{P}$ parameters absolute error ($T_{x}$, $T_{y}$, $T_{z}$) for each wheel position. (**a**): $T_{z}$ parameter fixed at 40 cm; (**b**): $T_{z}$ parameter fixed at 60 cm. Statistical significance is indicated using symbols: *ns* for not statistically significant, * for p<0.005, ** for p<0.001, and *** for p<0.0001.

The results of this test are reported in Figure 7 and Figure 8. Figure 7a,b show the mean absolute errors of the $CtP_{A}$ parameters at fixed working distances $T_{z}=40$ cm and $T_{z}=60$ cm, respectively. Figure 8a,b show the corresponding mean absolute errors for the $CtP_{P}$ parameters at the same distances.

Additionally, to assess whether the observed changes in variability with viewing angle are statistically meaningful, Levene’s test for equality of variances was applied to the error distributions. The statistical analysis was performed separately for the two working distances ($T_{z}=40\;\mathrm{cm}$ and $T_{z}=60\;\mathrm{cm}$), since they represent distinct experimental conditions and were evaluated independently. Within each working distance, the variance at the frontal configuration (0°) was compared against the variance at the two extreme viewpoints (−60° and +60°) for each parameter (φ, ζ, $T_{x}$, $T_{y}$, $T_{z}$). This resulted in 10 variance comparisons per working distance. Again, Levene’s test was selected because systematic effects can influence the mean absolute error, whereas the variance directly reflects repeatability and intrinsic measurement noise. For each distance-specific family of tests, a Bonferroni correction was applied to control for multiple comparisons, setting the corrected significance level to $\alpha_{B}=0.05/10=0.005$.

Across viewing angles, the mean absolute error on φ is minimal in the frontal configuration ($\varphi_{\mathrm{GT}}=0{}^{\circ}$) and increases progressively at more oblique views. Conversely, the standard deviation (error bars) is highest at $\varphi_{\mathrm{GT}}=0{}^{\circ}$ and decreases as the viewing angle departs from frontal, indicating a trade-off between accuracy (lower mean absolute error) and precision (lower variability). A similar behavior is observed for the in-plane rotation ζ. Comparing working distances, moving from $T_{z}=40\;\mathrm{cm}$ to $T_{z}=60\;\mathrm{cm}$ yields lower mean absolute errors (lower bars) but larger standard deviations (higher error bars) for both φ and ζ. Notably, the angle-dependent trend in variability is preserved at both distances, with the largest standard deviations near the frontal configuration and decreasing variability at increasing obliquity. To complement these qualitative trends, Levene’s test consistently indicated significant variance differences for the rotation parameters: the variability at 0° was significantly different from that at both −60° and +60° for φ and ζ. This statistically supports the observed decrease in standard deviation as the viewing angle departs from the frontal configuration. In contrast, translation parameters showed no significant variance differences in almost all comparisons, with the only exception of $T_{x}$ in the 0° vs. 60° comparison. Overall, this suggests that at $T_{z}=40\;\mathrm{cm}$ the viewing-angle dependence primarily affects the repeatability of rotation estimation, while translation variability remains largely stable across angles. For $T_{z}=60\;\mathrm{cm}$, a different pattern emerged. Variance differences remained significant for most rotation comparisons, with the exception of the φ comparison between 0° and −60°, which was not significant. Despite this isolated non-significant case, the overall trend still supports reduced variability at oblique angles. For translation parameters, variance differences were significant in most comparisons, except for the $T_{z}$ comparison between 0° and +60°. This broader occurrence of significant variance changes in $CtP_{P}$ at the larger working distance may indicate that increased measurement noise at 60 cm makes translation repeatability more sensitive to viewpoint, compared to the 40 cm condition.

### 3.3. Influence of Distance on Pose Estimation

**Figure 9:**
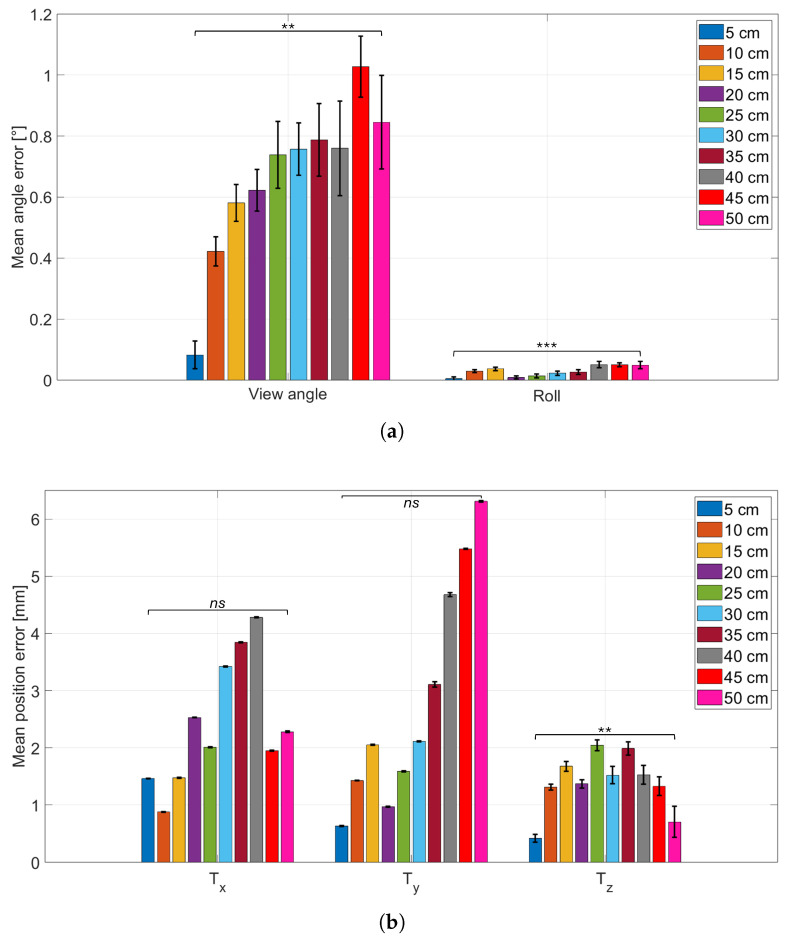
(**a**): $CtP_{A}$ parameters absolute error (bar height; viewing angle φ and roll ζ) with standard deviation (error bars) for each camera-to-ChArUco distance. (**b**): $CtP_{P}$ parameters mean absolute error (bar height; $T_{x}$, $T_{y}$, $T_{z}$) with standard deviation (error bars) across repeated measurements, for each camera-to-ChArUco working distance. Statistical significance is indicated using symbols: *ns* for not statistically significant, ** for p<0.0025, and *** for p<0.00025.

The results of this test are presented in Figure 9. In Figure 9a, the average errors related to $CtP_{A}$ parameters are presented, while in Figure 9b, the average errors related to $CtP_{P}$ parameters are presented.

To assess whether the observed changes in variability with distance are statistically meaningful, Levene’s test for equality of variances was applied to the error distributions. The variance at the lower working distance ($T_{z}=5\;\mathrm{cm}$) was compared against the variance at the higher working distance ($T_{z}=50\;\mathrm{cm}$) for each parameter (φ, ζ, $T_{x}$, $T_{y}$, $T_{z}$). Since this resulted in five variance comparisons, a Bonferroni correction was applied, setting the corrected significance level to $\alpha_{B}=0.05/5=0.01$.

Levene’s test indicates significantly different variances for the rotation parameters (φ and ζ) when comparing $T_{z}=5\;\mathrm{cm}$ to $T_{z}=50\;\mathrm{cm}$, consistent with a distance-dependent change in repeatability. This behavior may reflect increased sensitivity to pixel-level noise at larger working distances, where the board occupies fewer pixels and corner localization becomes less stable; alternatively, it may also be influenced by uncertainty in the distance-setting procedure. For the translation parameters, $T_{x}$ and $T_{y}$ do not show significant variance differences across the two distances, suggesting that their repeatability remains largely stable in the tested range. By contrast, $T_{z}$ exhibits a significant variance difference, supporting the observation that depth estimation becomes less repeatable at larger camera-to-board distances. Overall, these results indicate that increasing working distance primarily reduces the repeatability of $T_{z}$ estimation, even when the average agreement with ground truth remains comparable.

## 4. Discussion

The present CT-motivated benchtop study shows that camera–ChArUco geometry has distinct and sometimes opposing effects on pose-estimation accuracy (bias with respect to ground truth) and precision (repeatability). In particular, cyclic repositioning does not measurably increase variability compared to repeated estimates at fixed pose, viewing angle induces an accuracy–precision trade-off in rotation estimation, and increasing working distance reduces repeatability, especially for depth-related components.

The first objective was to verify whether repeated repositioning introduces additional variability beyond intrinsic pose-estimation noise. This hypothesis was not supported: variance-based testing did not provide evidence of increased variability under cyclic acquisitions after multiple-comparison correction. This result is relevant for CT-motivated, projection-wise tracking, where one estimate per view is delivered while the relative geometry changes between successive projections. In standing equine CT, motion mitigation has been mainly pursued via mechanical restraint and protocol choices rather than software-based projection-wise correction [12,13,14,15,16]. At the same time, recent CT research has proposed image-driven and learning-based motion compensation approaches that estimate motion directly from projections or reconstructed volumes [23,24,25]. The present finding complements these directions by supporting the feasibility of stable, view-by-view external sampling as an additional information source in motion-aware workflows.

The second objective was to characterize how viewpoint affects pose-estimation performance. Rotation estimation exhibits a consistent trade-off: frontal views yield lower mean absolute error but higher variability, whereas more oblique views reduce variability at the cost of larger mean error. Recent fiducial-marker studies confirm that challenging viewpoints and adverse imaging conditions are key failure modes: deep-learning-based detectors have been introduced to improve robustness under difficult illumination [26], and multi-marker strategies with outlier rejection have been shown to reduce both mean errors and variability, especially at large tilt angles [27]. Benchmarking across different cameras and setups further reports that pose stability can vary materially with sensing hardware and geometry [28]. In the standing equine CT context, where residual postural sway can lead to view-inconsistent motion and image artefacts, precision (low frame-to-frame jitter) can be a critical limiting factor for motion compensation pipelines. The present results highlight that a geometry optimized for minimal bias (strictly frontal view) may not be optimal when repeatability is prioritized.

The third objective was to evaluate the effect of working distance. Increasing the camera-to-board distance is associated with reduced repeatability, most markedly affecting depth-related components. A plausible mechanism is the reduced board footprint in the image at larger distances, which makes corner localization more sensitive to pixel-level noise. This observation is consistent with prior characterizations of low-cost RGB/RGB-D sensing for motion tracking [20,21,22] and aligns with recent fiducial-based camera-positioning analyses emphasizing the role of marker image coverage and viewing geometry in pose quality [33]. For CT-motivated deployments, this implies that distance should be selected to keep the target sufficiently resolved in the image while satisfying practical constraints of the scanner environment.

Although the present work is not a clinical validation study, the observed trends can inform pre-clinical configuration of an external tracking setup before in-scanner and in-vivo evaluation. In standing equine CT, where external tracking may support projection-wise motion compensation, the following implications arise:

- A strictly frontal configuration may minimize bias but can maximize jitter; therefore, a moderately oblique nominal viewpoint may be preferable when repeatability is prioritized.
- Excessive working distances should be avoided to preserve repeatable depth estimation.
- The board should occupy a sufficiently large image region to stabilize corner localization.
- Rigid mounting and minimization of occlusions/illumination changes remain practical requirements in the clinical workflow.

Several limitations should be acknowledged. Experiments were conducted under controlled laboratory conditions using a single board layout and a single RGB sensor; real deployments may involve occlusions, specularities, illumination changes, and multi-axis motion. Only limited degrees of freedom were explored (single-axis rotation and axial distance changes). In addition, repeated CT acquisitions solely for marker-placement optimization would increase radiation exposure without clinical benefit; therefore, the present study focuses on benchtop characterization to reduce the need for iterative animal experiments. Finally, the analysis focuses on pose-estimation metrics rather than end-to-end image-quality outcomes.

Future work should extend the study to multi-axis trajectories and validate the proposed setup choices in realistic scanner environments. When ethically justified (e.g., clinically indicated scans), in-vivo validation should quantify the impact of the chosen geometry on motion-compensated reconstructions and image quality (e.g., sharpness and streak reduction). Additional extensions include evaluating different ChArUco scales/layouts, multi-camera fusion, and the integration of RGB-D information where available.

## 5. Conclusions

This CT-motivated benchtop study provides pre-clinical guidance on how camera–ChArUco geometry impacts pose-estimation accuracy and repeatability in external tracking setups relevant to standing equine CT. The results highlight that viewpoint and working distance can affect bias and jitter differently, motivating geometry choices based on the relative importance of accuracy versus repeatability in motion-compensation pipelines. Future work will validate these setup choices in realistic scanner environments and, when ethically justified, in vivo using clinically indicated scans, including end-to-end evaluation on motion-compensated reconstructions.

## Figures and Tables

**Table 1:** Main characteristics of the RGB module of the Intel D435 3D camera.

Frame resolution	1920×1080
Frame rate	30 fps
Sensor technology	Rolling shutter
Sensor FOV (H × V)	69° × 42°
Sensor resolution	2 MP

## Data Availability

The data presented in this study are available on request from the corresponding author due to legal reasons.
